# Oocyte degeneration in a cohort adversely affects clinical outcomes in conventional IVF cycles: a propensity score matching study

**DOI:** 10.3389/fendo.2023.1164371

**Published:** 2023-05-19

**Authors:** Lanlan Liu, Xiaoming Jiang, Zhenfang Liu, Jinghua Chen, Chao Yang, Kaijie Chen, Xiaolian Yang, Jiali Cai, Jianzhi Ren

**Affiliations:** ^1^ Reproductive Medicine Center, The Affiliated Chenggong Hospital of Xiamen University, Xiamen, China; ^2^ Medical College, Xiamen University, Xiamen, China

**Keywords:** oocyte degeneration, conventional IVF, embryo development potential, clinical outcome, cumulative live birth

## Abstract

**Background:**

Oocyte degeneration was mostly described in intracytoplasmic sperm injection (ICSI) cycles; there is no report showing the relationship between oocyte degeneration and clinical outcomes in conventional *in vitro* fertilization (IVF) cycles. This retrospective study using the propensity score (PS) matching method aimed to explore whether the presence of oocyte degeneration in conventional IVF cycles would affect the sibling embryo development potential and clinical outcomes.

**Methods:**

Patients with at least one oocyte degenerated after short-term insemination and stripping were defined as the degeneration (DEG) group, while patients with no oocyte degenerated were defined as the non-degeneration (NONDEG) group. The PS matching method was used to control for potential confounding factors, and a multivariate logistic regression analysis was made to evaluate whether the presence of oocyte degeneration would affect the cumulative live birth rate (CLBR).

**Results:**

After PS matching, basic characteristics were similar between the two groups, oocyte yield was significantly higher in the DEG group than the NON-DEG group (P < 0.05), mature oocyte number, 2 pronuclear (2PN) embryo number, 2PN embryo clearage rate, “slow” embryo number, “accelerated” embryo number, rate of cycles with total day 3 embryo extended culture, number of frozen embryo transfer (FET) cycles, transferred embryo stage, transferred embryo number, and live birth rate in fresh embryo transfer cycles were all similar between the two groups (P > 0.05), but the 2PN fertilization rate, available embryo number, high-quality embryo number, “normal” embryo number, frozen embryo number, blastocyst formation rate, and no available embryo cycle rate were all significantly lower in the DEG group than the NON-DEG group (P < 0.05). The cumulative live birth rate was also significantly lower in the DEG group than in the NON-DEG group (70.2% vs. 74.0%, P = 0.0019). Multivariate logistic regression analysis further demonstrated that the presence of oocyte degeneration in conventional IVF cycles adversely affects the CLBR both before (OR = 0.83, 95% CI: 0.75–0.92) and after (OR = 0.82, 95% CI: 0.72–0.93) PS matching.

**Conclusion:**

Our findings together revealed that the presence of oocyte degeneration in a cohort of oocytes may adversely affect subsequent embryo development potential and clinical outcomes in conventional IVF cycles.

## Introduction

Oocyte degeneration is a common phenomenon in both conventional *in vitro* fertilization (IVF) and intracytoplasmic sperm injection (ICSI) cycles, but it is mostly described in ICSI cycles (1, 2). The presence of degenerated oocytes may be an indicator of either laboratory performance or oocyte quality (2). It is proposed that the presence of degenerated oocyte or empty zona pellucida at ovum pick-up (OPU) before ICSI correlates with the quality of the entire oocyte cohort (3–5). However, several authors argued that oocyte degeneration in ICSI cycles is neither technician nor physician dependent but reflects the inherent oocyte quality, as the changes in the ICSI technician or the stripping technician were not associated with the oocyte degeneration rate (1). Nevertheless, ICSI remained a strong confounder in the reported studies and the performance of ICSI may vary among embryologists and clinics. The random fluctuation of ICSI performance may also mask the association between oocyte quality and oocyte degeneration.

The invasive procedure of ICSI is absent in conventional IVF cycles, which provided an opportunity to evaluate the association between oocyte quality and oocyte degeneration without confounding ICSI performance. Oocyte degeneration could be confirmed during oocyte denudation or at the time of fertilization assessment with retracted and/or darkened ooplasm or without zona pellucida the next day after OPU during conventional IVF cycles (1, 6). However, data regarding oocyte degeneration in conventional IVF cycles remained very limited. Only one study reported a negative association between oocyte degeneration and the IVF outcome (7). However, both conventional IVF cycles and ICSI cycles were all included in that study. Meanwhile, oocyte degeneration was assessed at the time of OUP, as we know, oocytes were surrounded by layers of granulosa cells after retrieval. Oocyte degeneration like retracted ooplasm within the zona pellucid is difficult to confirm at the time of OPU, and cumulative live birth was also not studied in that study.

Most of the previous studies regarding oocyte degeneration associated the degeneration rate with the implantation rate following fresh embryo transfer (7, 8). These study designs may lead to selection bias as they could not include certain patients such as patients with exceeded responses or planning a freeze-all procedure. If oocyte degeneration correlates with the quality of the entire oocyte cohort, the cumulative outcomes of the oocyte cohort should be a better measure than the outcome of a single transfer.

In this study, we aimed to investigate whether the presence of oocyte degeneration in conventional IVF cycles on the OPU day would affect the sibling embryo quality and clinical outcomes in a retrospective cohort including 16,823 conventional IVF cycles, of which 2,776 cycles presented at least one oocyte degenerated. A propensity score (PS) matching method was used to account for comparability between the two groups by balancing the biases and confounders, and multivariate logistic regression was carried out to confirm the findings.

## Materials and methods

Institutional review board approval for this study was obtained from the Ethical Committee of Medical College Xiamen University. Informed consent was not necessary because this retrospective research was based on non-identifiable records.

### Study subjects

This retrospective study was performed at the reproductive medicine center of The Affiliated Chenggong Hospital of Xiamen University. Patients who underwent conventional IVF treatment from 2013 to 2019 were enrolled and retrospectively analyzed. The exclusion criteria were as follows: (a) patients proposed to take IVF treatment but eventually underwent early rescue ICSI treatment because less than 50% of mature oocytes excluded their second poly body. (b) Patients got no live birth but still had frozen embryos 2 years later after oocyte collection in one OPU cycle. A total of 14,665 patients who took 16,823 conventional IVF cycles were included, of which 2,776 cycles presented at least one oocyte degenerated after short-term insemination and denuding on the OPU day, and 12,921 cycles took fresh embryo transfer. Cycles with at least one oocyte degenerated were defined as the degeneration group (DEG Group), and cycles with no oocytes degenerated were defined as the non-degeneration group (NON DEG Group).

### Laboratory procedures and embryo assessment

Most patients in our center used a Gonadotropin-releasing hormone (GnRH) agonist or an antagonist protocol as we previously described (9, 10); some patients with advanced age or poor ovarian reserve used “other protocols” like the mild stimulation protocol, natural cycle, or luteal-phase stimulation protocol. Ovarian response was monitored using transvaginal ultrasound measurements of follicular growth and the serum E_2_ level every 1–3 days. Oocyte retrieval was performed using transvaginal ultrasound, and a 17 G needle (Cook Medical) was used for oocyte pick-up.

Insemination was carried out using conventional IVF, cumulus–oocyte complexes were incubated with approximately 1.5–3 × 10^5^ progressively motile spermatozoa in fertilization culture medium (K-SIFM, Cook) for approximately 5 h, and then oocytes were denuded for the observation of the second poly body. Patients with more than 50% mature oocytes excluding the second poly body underwent conventional IVF treatment, while early rescue ICSI was performed on patients with less than 50% mature oocytes excluding the second poly body 6 h after insemination. Early rescue ICSI patients were excluded in this study. Considering oocyte degeneration definition in ICSI cycles (1, 2), degeneration oocytes in this study were defined as those broken to pieces during stripping or being noted by retracted and/or darkened ooplasm or with empty zona pellucid after denudation for the observation of the second poly body after stripping.

Embryos were cultured in single droplets after fertilization, and oocytes and embryos were cultured in COOK series medium (KSIFM, KSICM, or KSIBM, Cook Medical, Bloomington, IN) in traditional incubators (C200, Labotech, Gottingen, Germany) at 37°C, 6% CO_2_, and 5% O_2_. Fertilization was checked approximately 17–18 h post-insemination under an inverted microscope. Day 3 embryo assessment and grading system were based on the number of embryo blastomeres, fragmentation, and symmetry; 2PN embryos on day 3 were assessed and divided into four different grades according to the ASEBIR embryo assessment criteria (6). Grade 1 and grade 2 embryos were considered as high-quality embryos; grade 1, grade 2, and grade 3 embryos were considered as available embryos, while grade 4 embryos, arrested embryos, and embryos with all blastomere degenerated or lysed were usually deserted. Meanwhile, as for embryo development kinetics, according to the Istanbul consensus (6), “normal” embryos have 7–9 cells on day 3, “slow” embryos have 6 or fewer cells on day 3, and “accelerated” embryos have >9 cells on day 3. For blastocyst assessment, we used the Gardner grading system (11), and we previously described it in detail (9). Blastocysts with poor morphology scores (≤CC) or low expansion grades (grade 1–2) were not cryopreserved or transferred.

A vitrification protocol was used for embryo cryopreservation, which employed 15% dimethyl sulfoxide, 15% ethylene glycol, and 0.6 M sucrose as cryoprotectants. For embryo thawing, embryos were directly immersed into a thawing solution (TS) containing 1 M sucrose at 37°C for 1 min. Then, they were sequentially incubated in each of the following solutions for 3 min: 0.5 M sucrose, 0.25 M sucrose, and sucrose-free TS, and embryos were placed in blastocyst culture medium (K-SIBM, COOK, IN, USA) and cultured normally.

Embryo transfer was performed using a COOK catheter (K-JETS-7019-SIVE, COOK, IN, USA) under trans-abdominal ultrasound guidance.

### Cumulative live birth calculation

The cumulative live birth rate (CLBR) was calculated in this study, and we used a short-term calculation method as previously described (12). The CLBR was presented as live birth episodes per woman per egg collection over a 2-year period to account for the first live birth.

### Statistical analysis

Oocyte degeneration in conventional IVF cycles occurs occasionally, and there were differences in basic characteristics between the DEG Group and the NON DEG Group; thus, we used the PS matching method to tackle potential confounders and selection biases in this study. PSs were calculated using logistic regression based on potential variables related to the outcomes. The variables included female age, male age, freeze-all strategy, ovarian stimulation protocol, infertility type, OPU order, the duration of infertility, whether complicated with polycystic ovary syndrome, whether complicated with endometriosis, whether complicated with tubal factor, female BMI, baseline hormone levels, antral follicle count (AFC), gonadotropin total dose, the length of stimulation, gonadotropin starting dose, E_2_ level on the hCG day, and survival oocyte number (retrieved oocyte number minus degenerated oocyte number). Considering degenerated oocytes decreased the survival oocyte number and may affect cumulative live birth, we take the survival oocyte number as a variable in the PS matching method. A one-to-one nearest neighbor matching method without replacement was performed to match data between the DEG group and the NON DEG group with a caliper width equal to 0.2. PS matching was performed by using the MatchIt package in R software (13). The cobalt package (14) was used to test the balance. Standard differences (D) were calculated to evaluate the balance of the distribution of the baseline characteristics between the two groups before and after PS matching. D < 0.1 was used as the threshold to indicate a negligible difference in the mean or prevalence of a covariate between exposure groups (15).

Data were presented as mean (SD) and median (first quartile and third quartile) for continuous variables and n (percentage) for categorical variables. Continuous variables were analyzed using the Wilcoxon test, and categorical variables were analyzed using the chi-square test or Fisher’s exact test; P < 0.05 was considered to be significant. All analyses were performed by using R statistic software 4.12 (16).

Multivariate logistic regression analysis was also performed to evaluate the association between oocyte degeneration presence and the probability of cumulative live birth, with adjustments made for important covariates and potential confounding factors.

We used generalized additive models (GAMs) to verify whether there is a dose-dependent association between degeneration and CLBR (17). In GAMs, the degree of degeneration and other continuous covariates were fitted as smooth terms. Because the smooth estimator only demonstrated the contribution of the covariate fitted to a given smooth function to the response variable, we plotted the degeneration against the prediction to demonstrate a dose response on a cumulative live birth.

## Results

In this study, 16,823 IVF cycles were enrolled and analyzed, 3,748 oocytes in 2,776 cycles were found degenerated after fertilization, the oocyte degeneration rate is 2.48% (3,748/151,047), and the degeneration cycle rate is 16.50% (2,776/16,823).

After PS matching, 2,776 cycles in the NON DEG group were matched by their counterparts in the DEG group. Basic characteristics before and after PS matching are shown in **Table 1**. Before matching (left panel), significant differences were observed between the DEG group and the NON DEG group, including female age, male age, embryo freeze-all cycle rate, the rate of the GnRH agonist protocol, basal FSH level, AFC, the length of stimulation, E_2_ level on the hCG day, and survival oocyte number (D > 0.1). After matching (right panel), all the baseline characteristics became very comparable between the two groups (D < 0.1). Distributions of the PSs before and after PS matching are shown in **Supplementary Figure S1**.

**Table 1:** Baseline characteristics of the degeneration (DEG) group and non-degeneration (NON DEG) group before and after propensity score (PS) matching.

Variables	Before matching			After matching		
	NON DEG	DEG	*D	NON DEG	DEG	*D
	(N = 14,047)	(N = 2,776)		(N = 2,776)	(N = 2,776)	
Female’s age, year			-0.1717			0.0469
Mean (SD)	31.9 (4.62)	31.2 (4.25)		31.0 (4.21)	31.2 (4.25)	
Median [Min, Max]	31.0 [20.0, 48.0]	31.0 [20.0, 45.0]		30.0 [20.0, 46.0]	31.0 [20.0, 45.0]	
Male’s age, year			-0.1193			0.0128
Mean (SD)	33.6 (5.17)	33.0 (4.93)		33.0 (4.90)	33.0 (4.93)	
Median [Min, Max]	33.0 [22.0, 60.0]	32.0 [22.0, 59.0]		32.0 [22.0, 56.0]	32.0 [22.0, 59.0]	
**Freeze-all cycle (%)**	2,300 (16.4%)	818 (29.5%)	**0.1309**	801 (28.9%)	818 (29.5%)	0.0061
Stimulation protocols						
GnRH agonist (%)	9,178 (65.3%)	2,139 (77.1%)	**0.1172**	2,165 (78.0%)	2,139 (77.1%)	-0.0094
GnRH antagonist (%)	2,856 (20.3%)	375 (13.5%)	-0.0682	367 (13.2%)	375 (13.5%)	0.0029
Other protocols (%)	2,013 (14.3%)	262 (9.4%)	-0.0489	244 (8.8%)	262 (9.4%)	0.0065
**Primary infertility (%)**	5,569 (39.6%)	1,201 (43.3%)	-0.0362	1,200 (43.2%)	1,201 (43.3%)	-0.0004
OPU order						
1	11,358 (80.9%)	2,358 (84.9%)	0.0409	2,366 (85.2%)	2,358 (84.9%)	-0.0029
2	2,027 (14.4%)	344 (12.4%)	-0.0204	348 (12.5%)	344 (12.4%)	-0.0014
≧3	662 (4.7%)	74 (2.7%)	-0.0205	62 (2.2%)	74 (2.7%)	0.0043
**Duration of infertility, year**			-0.0015			0.0151
Mean (SD)	4.21 (3.13)	4.21 (3.05)		4.16 (3.00)	4.21 (3.05)	
Median [Min, Max]	3.50 [0.04, 31.0]	3.50 [0.10, 23.0]		3.50 [0.10, 25.0]	3.50 [0.10, 23.0]	
**PCOs (%)**	915 (6.5%)	203 (7.3%)	0.008	201 (7.2%)	203 (7.3%)	0.0007
**Endometriosis (%)**	1,744 (12.4%)	347 (12.5%)	0.0008	341 (12.3%)	347 (12.5%)	0.0022
**Tubal factor (%)**	10,452 (74.4%)	2,048 (73.8%)	-0.0063	2,058 (74.1%)	2,048 (73.8%)	-0.0036
**Female BMI, kg/cm^2^**			-0.0836			-0.0279
Mean (SD)	21.2 (2.26)	21.0 (2.26)		21.1 (2.29)	21.0 (2.26)	
Median [Min, Max]	21.2 [13.5, 46.8]	21.0 [15.5, 46.2]		21.0 [14.3, 46.2]	21.0 [15.5, 46.2]	
**Basal FSH, IU/l**			**-0.1161**			0.0245
Mean (SD)	8.20 (2.99)	7.88 (2.79)		7.81 (2.72)	7.88 (2.79)	
Median [Min, Max]	7.50 [0.05, 41.3]	7.25 [0.10, 41.5]		7.16 [0.05, 41.3]	7.25 [0.10, 41.5]	
**Basal LH, IU/l**			0.0679			-0.0146
Mean (SD)	4.84 (2.93)	5.05 (3.07)		5.09 (3.11)	5.05 (3.07)	
Median [Min, Max]	4.24 [0.01, 51.6]	4.37 [0.05, 38.6]		4.45 [0.01, 30.9]	4.37 [0.05, 38.6]	
**Basal PRL, ng/ml**			0.0423			-0.0122
Mean (SD)	15.6 (8.47)	16.1 (10.4)		16.2 (9.14)	16.1 (10.4)	
Median [Min, Max]	14.1 [0.19, 162]	14.1 [0.17, 298]		14.3 [0.22, 162]	14.1 [0.17, 298]	
**Basal E_2_, pg/ml**			-0.0356			0.019
Mean (SD)	45.9 (31.1)	44.9 (25.7)		44.5 (23.5)	44.9 (25.7)	
Median [Min, Max]	41.0 [0.03, 747]	41.0 [0.10, 499]		41.0 [2.0, 321]	41.0 [0.10, 499]	
**Antral follicle count**			**0.1892**			-0.0156
Mean (SD)	10.1 (5.43)	11.1 (5.41)		11.2 (5.40)	11.1 (5.41)	
Median [Min, Max]	9.00 [1.00, 32.0]	10.0 [1.0, 30.0]		10.0 [1.0, 30.0]	10.0 [1.0, 30.0]	
**Total dose of GN, IU**			0.0462			-0.0088
Mean (SD)	2,280 (700)	2,310 (643)		2,310 (648)	2,310 (643)	
Median [Min, Max]	2,250 [0, 8,480]	2,250 [0, 8,100]		2,250 [0, 8,210]	2,250 [0, 8,100]	
**Length of stimulation, day**			**0.1523**			-0.0139
Mean (SD)	11.1 (3.07)	11.5 (2.55)		11.5 (2.74)	11.5 (2.55)	
Median [Min, Max]	11.0 [0, 19.0]	11.0 [0, 18.0]		11.0 [0, 17.0]	11.0 [0, 17.0]	
**GN starting dose, IU**			-0.0568			-0.0042
Mean (SD)	199 (43.1)	196 (39.7)		197 (38.7)	196 (39.7)	
Median [Min, Max]	225 [0, 300]	225 [0, 300]		225 [0, 300]	225 [0, 300]	
**E_2_ level of hCG day**			**0.3415**			0.0187
Mean (SD)	2,980 (2,450)	3,910 (2730)		3,860 (2,720)	3,910 (2,730)	
Median [Min, Max]	2,430 [0, 105,000]	3,520 [0, 45,500]		3,490 [0, 43,800]	3,520 [0, 45,500]	
**Survival oocyte number**			**0.4405**			0.0041
Mean (SD)	8.29 (5.59)	11.1 (6.47)		11.1 (6.50)	11.1 (6.47)	
Median [Min, Max]	7.00 [1.00, 52.0]	10.0 [1.00, 45.0]		10.0 [1.00, 52.0]	10.0 [1.00, 45.0]	

Data were presented as mean ± SD and median [first quartile, third quartile] for continuous variables and n (percentage) for categorical variables. *D: Standardized difference. The absolute value of D ≥ 0.1 is printed in bold. The absolute value of D is less than 0.1, cohorts can be considered to be balanced with respect to the demographics being assessed. PCOS, polycystic ovarian syndrome; BMI, body mass index; FSH, follicle-stimulating hormone; LH, luteinizing hormone; PRL, prolactin; E_2_, estradiol; GN, gonadotropin.

**Table 2** shows the outcomes of the DEG group and the NON DEG group before and after PS matching. After PS matching, oocyte yield was significantly higher in the DEG group than the NON DEG group (*P* < 0.05), mature oocyte number, fertilization oocyte number, 2PN embryo number, 2PN embryo clearage rate, “slow” embryo number, “accelerated” embryo number, the rate of cycles with total day 3 extended embryo culture, the number of FET cycles, transferred embryo stage, transferred embryo number, and live birth rate in fresh cycles were all similar between the two groups (*P* > 0.05), but the 2PN fertilization rate, both on the basis of oocyte yield (2PN number/oocyte yield *100%) as shown in **Table 2** and on the basis of the survival oocyte number (2PN number/survival oocyte number *100%, 65.2 ± 21.5 vs. 62.3 ± 22.6, P < 0.001, in matched cohort) were all significantly lower in the DEG group than the NON DEG group (*P* < 0.05). The available embryo number, high-quality embryo number, “normal” embryo number with seven to nine cells on day 3, frozen embryo number, blastocyst formation rate, and no available embryo cycle rate were all significantly lower in the DEG group than the NON DEG group (*P* < 0.05). These findings revealed that the presence of oocyte degeneration in a cohort of oocytes may affect subsequent embryo development potential. The cumulative live birth rate was also significantly lower in the DEG group than the NON DEG group (70.2% vs. 74.0%, *P* = 0.0019) after PS matching.

**Table 2:** Clinical outcomes of DEG group and NON DEG group before and after PS matching.

Variables	Before matching			After matching		
	NON DEG	DEG	*P*	NON DEG	DEG	P
	(N = 14,047)	(N = 2,776)		(N = 2,776)	(N = 2,776)	
**Oocyte yield**			<0.001			<0.001
Mean (SD)	8.29 (5.59)	12.5 (6.56)		11.1 (6.50)	12.5 (6.56)	
Median [Min, Max]	7.00 [1.00, 52.0]	12.0 [2.0, 46.0]		10.0 [1.00, 52.0]	12.0 [2.00, 46.0]	
**Mature oocyte number**			<0.001			0.874
Mean (SD)	7.54 (5.19)	10.0 (6.04)		10.1 (6.06)	10.0 (6.04)	
Median [Min, Max]	6.00 [1.00, 46.0]	9.00 [1.0, 44.0]		9.00 [1.00, 45.0]	9.00 [1.00, 44.0]	
**Fertilization oocyte number**			<0.001			0.218
Mean (SD)	6.94 (4.92)	9.10 (5.72)		9.28 (5.77)	9.10 (5.72)	
Median [Min, Max]	6.00 [0, 41.0]	8.00 [0, 39.0]		8.00 [0, 39.0]	8.00 [0, 39.0]	
**2PN embryo number**			<0.001			0.0723
Mean (SD)	5.32 (3.94)	6.93 (4.67)		7.12 (4.59)	6.93 (4.67)	
Median [Min, Max]	4.00 [0, 27.0]	6.00 [0, 36.0]		6.00 [0, 27.0]	6.00 [0, 36.0]	
**Normal fertilization rate (%)**			<0.001			<0.001
Mean (SD)	65.1 (24.8)	53.6 (19.5)		65.2 (21.5)	53.6 (19.5)	
Median [Min, Max]	66.7 [0, 100]	56.1 [0, 94.4]		66.7 [0, 100]	56.1 [0, 94.4]	
**Cleavage rate of 2PN embryo (%)**			0.017			0.0679
Mean (SD)	94.2 (20.7)	94.8 (18.5)		96.5 (14.1)	94.8 (18.5)	
Median [Min, Max]	100 [0, 100]	100 [0, 100]		100 [0, 100]	100 [0, 100]	
**Available embryo number**			<0.001			0.0452
Mean (SD)	4.78 (3.84)	6.25 (4.49)		6.46 (4.40)	6.25 (4.49)	
Median [Min, Max]	4.00 [0, 27.0]	6.00 [0, 34.0]		6.00 [0, 27.0]	6.00 [0, 34.0]	
**High-quality embryo number**			<0.001			0.0064
Mean (SD)	3.23 (2.95)	4.11 (3.51)		4.32 (3.45)	4.11 (3.51)	
Median [Min, Max]	3.00 [0, 20.0]	3.00 [0, 28.0]		4.00 [0, 20.0]	3.00 [0, 28.0]	
**“Slow” embryo number**			<0.001			0.219
Mean (SD)	2.32 (2.39)	3.08 (2.95)		3.16 (2.94)	3.08 (2.95)	
Median [Min, Max]	2.00 [0, 23.0]	2.00 [0, 22.0]		2.00 [0, 23.0]	2.00 [0, 22.0]	
**“Normal” embryo number**			<0.001			0.0132
Mean (SD)	3.43 (2.99)	4.29 (3.45)		4.47 (3.38)	4.29 (3.45)	
Median [Min, Max]	3.00 [0, 29.0]	4.00 [0, 24.0]		4.00 [0, 21.0]	4.00 [0, 24.0]	
**“Accelerated” embryo number**			<0.001			0.514
Mean (SD)	0.560 (1.10)	0.709 (1.30)		0.724 (1.28)	0.709 (1.30)	
Median [Min, Max]	0 [0, 13.0]	0 [0, 14.0]		0 [0, 10.0]	0 [0, 14.0]	
**Percentage of cycles with total day 3 embryo extended culture (%)**	10,327 (73.5%)	1,737 (62.6%)	<0.001	1,762 (63.5%)	1,737 (62.6%)	0.505
**Blastocyst formation rate (%)**			0.271			<0.001
Mean (SD)	52.5 (31.5)	54.7 (27.9)		59.6 (25.2)	54.7 (27.9)	
Median [Min, Max]	58.3 [0, 100]	58.8 [0, 100]		62.5 [0, 100]	58.8 [0, 100]	
**Frozen embryo number**			<0.001			0.0181
Mean (SD)	2.40 (2.73)	3.32 (3.25)		3.52 (3.32)	3.32 (3.25)	
Median [Min, Max]	2.00 [0, 20.0]	3.00 [0, 26.0]		3.00 [0, 20.0]	3.00 [0, 26.0]	
**No available embryo cycle rate (%)**	668 (4.8%)	116 (4.2%)	0.205	59 (2.1%)	116 (4.2%)	<0.001
**Number of FET cycle**			<0.001			0.282
Mean (SD)	0.568 (0.820)	0.718 (0.920)		0.754 (0.954)	0.718 (0.920)	
Median [Min, Max]	0 [0, 6.00]	0 [0, 6.00]		0 [0, 5.00]	0 [0, 6.00]	
**Transferred embryo stage in fresh cycle**			<0.001			0.166
day 2	1,047 (9.5%)	142 (7.7%)		121 (6.3%)	142 (7.7%)	
day 3	8,765 (79.1%)	1,442 (78.3%)		1,503 (78.4%)	1,442 (78.3%)	
day 5	1,267 (11.4%)	258 (14.0%)		292 (15.2%)	258 (14.0%)	
**Transferred embryo number in fresh cycle**			0.553			0.362
1	4,071 (36.7%)	654 (35.5%)		690 (36.0%)	654 (35.5%)	
2	6,818 (61.5%)	1,158 (62.9%)		1,205 (62.9%)	1,158 (62.9%)	
3	190 (1.7%)	30 (1.6%)		21 (1.1%)	30 (1.6%)	
**Fresh cycle LB rate (%)**	5,504 (49.7%)	1,001 (54.3%)	<0.001	1,046 (54.6%)	1,001 (54.3%)	0.904
**Cumulative LB rate (%)**	9,028 (64.3%)	1,949 (70.2%)	<0.001	2,054 (74.0%)	1,949 (70.2%)	0.0019

Data were presented as mean ± SD and median [first quartile, third quartile] for continuous variables and n (percentage) for categorical variables. PN, pronuclei; FET, frozen embryo transfer; LB, live birth; “slow” embryo, embryos with 6 or fewer cell numbers on day 3; “normal” embryo, embryos with 7–9 cell numbers on day 3; “accelerated” embryo, embryos with >9 cell number on day 3.

**Figure 1:**
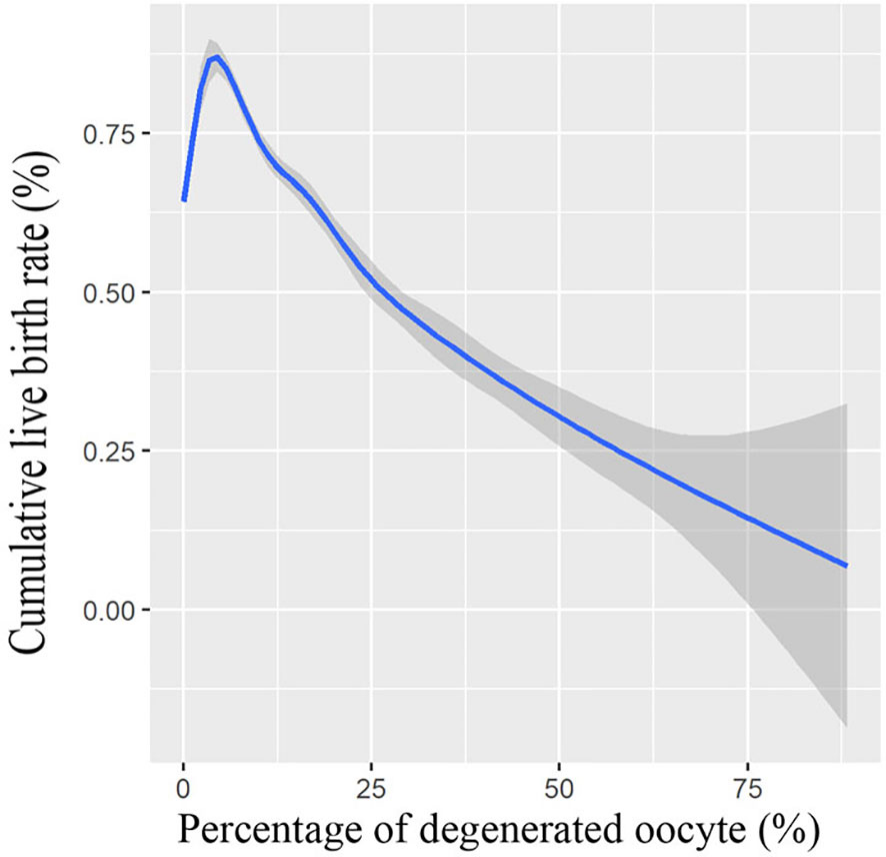
Association between cumulative live birth rate and percentage of degenerated oocytes. Shade indicates confident intervals. Model was fitted with GAM, adjusted for female age, male age, freeze-all cycle rate, ovarian stimulation protocol, primary infertility rate, OPU order, duration of infertility, polycystic ovary syndrome patient rate, endometriosis patient rate, tubal factor patient rate, female BMI, baseline hormone levels, antral follicle count (AFC), the total dose of gonadotropin, length of stimulation, gonadotropin starting dose, E2 level on hCG day, and survival oocyte number (retrieved oocyte number minus degenerated oocyte number).

We also conducted a multivariate logistic regression analysis, with adjustments made for important covariates and potential confounding factors; logistic regression analysis results in **Table 3** further demonstrated that the presence of oocyte degeneration in conventional IVF cycles adversely affect the cumulative live birth rate both before (OR = 0.83, 95% CI: 0.75–0.92) and after PS matching (OR = 0.82, 95% CI: 0.72–0.93). Meanwhile, with the GMA method, we found that the oocyte degeneration rate is negatively correlated with the cumulative live birth rate in an OPU cycle in a dose–response manner, results were shown in **Figure 1**.

**Table 3:** Multivariate logistic regression analysis for the cumulative live birth rate.

Variable	Category	Before matching			After matching		
		OR	95% CI	*P*	OR	95% CI	*P*
Oocyte degeneration	Degeneration group vs. control group	0.83	0.75, 0.92	0.001	0.82	0.72, 0.93	<0.001
Female age	Per year increased	0.92	0.91, 0.93	<0.001	0.94	0.91, 0.96	<0.001
Male age	Per year increased	1	0.99, 1.01	0.8	1	0.98, 1.02	0.8
Stimulation protocol							
1	Antagonist vs. agonist protocol	0.62	0.54, 0.72	<0.001	0.53	0.40, 0.71	<0.001
2	Other protocols vs. agonist protocol	0.62	0.55, 0.69	<0.001	0.66	0.53, 0.83	0.003
Infertility type	Secondary infertility vs. primary infertility	1.12	1.03, 1.22	0.019	1.17	1.01, 1.36	0.028
OPU time							
Order 2 vs. 1	2 vs. 1	0.97	0.88, 1.08	>0.9	0.92	0.76, 1.11	0.532
Order 3 or more vs. 1	≥3 vs. 1	0.73	0.61, 0.87	0.008	0.61	0.41, 0.91	0.016
Duration of infertility	Per year increased	0.97	0.96, 0.98	<0.001	0.97	0.95, 0.99	0.041
PCOS	Yes vs. no	0.75	0.62, 0.91	0.007	0.79	0.56, 1.13	0.8
Endometriosis	Yes vs. no	1.11	0.99, 1.24	0.058	0.96	0.79, 1.18	0.8
Tubal factor	Yes vs. no	1.16	1.06, 1.26	0.003	1.15	0.98, 1.34	0.025
BMI	Per unit increased	1.01	0.99, 1.03	0.092	1.02	0.98, 1.05	0.025
Basal FSH	Per unit increased	0.97	0.96, 0.98	<0.001	0.96	0.93, 0.98	0.021
Basal LH	Per unit increased	1.01	0.99, 1.02	0.4	1.02	0.99, 1.05	0.3
Basal PRL	Per unit increased	1	1.00, 1.00	0.8	1	1.00, 1.01	0.3
Basal E_2_	Per unit increased	1	1.00, 1.00	0.2	1	1.00, 1.00	0.4
AFC	Per AFC increased	1.02	1.01, 1.03	<0.001	1.02	1.00, 1.03	0.047
The total dose of GN	Per 75 units increased	0.97	0.95, 0.98	<0.001	0.95	0.92, 0.98	0.001
Length of simulation	Per day increased	1.13	1.08, 1.18	<0.001	1.14	1.05, 1.25	0.011
GN starting dose	Per unit increased	1.22	1.07, 1.39	0.052	1.13	0.87, 1.49	0.2
E_2_ level on hCG day	Per 100 units increased	1.01	1.01, 1.02	<0.001	1.01	1.01, 1.01	<0.001
Survival oocyte number	Per oocyte increased	1.09	1.08, 1.10	<0.001	1.09	1.07, 1.10	<0.001
Freeze all or not	Freeze all cycle vs. fresh embryo transfer cycle	0.77	0.69, 0.86	<0.001	0.72	0.60, 0.86	<0.001

OPU, oocyte pick-up; PCOS, polycystic ovarian syndrome; BMI, body mass index; AFC, antral follicle count; FSH, follicle-stimulating hormone; LH, luteinizing hormone; PRL, prolactin; E_2_, estradiol; GN, gonadotropin; OR, odds ratio; CI, confidence interval.

## Discussion

To our best knowledge, this is the first study to investigate oocyte degeneration in conventional IVF cycles on the OPU day. Despite the surviving number of oocytes and the patient’s characteristics being comparable following PS matching, our results showed that the cumulative live birth rate of the completed cycles was significantly decreased in the DEG group than that in the non-DEG group, suggesting an overall decrease in the oocyte quality. It was also supported by the findings that the numbers of available embryos, high-quality embryos, and “normal” embryos with seven to nine cells on day 3 were all significantly lower in the DEG group than in the NON-DEG group. For patients who had all their embryos extended culture, the blastocyst formation rate was also decreased with the presence of oocyte degeneration. Taken together, these results revealed that the presence of oocyte degeneration in a cohort of oocytes in conventional IVF cycles may be an unfavorable predictor for subsequent embryo development potential and clinical outcomes.

In this study, we found that the percentage of conventional IVF cycles with oocyte degeneration on the OPU day is 16.50%, which is similar to previously reported at the time of OPU in both conventional IVF cycles and ICSI cycles (7). On the other hand, however, the oocyte degeneration rate among all oocytes retrieved was 2.48% in the present study, which is much lower than those reported in ICSI cycles (8). The difference might suggest the role of the invasive procedure of ICSI on the occurrence of oocyte degeneration.

In ICSI cycles with oocyte degeneration, several studies reported that oocyte degeneration was not an indicator of the live birth rate (8, 18), and Rosen et al. (1) concluded that “the remaining cohort of retrieved oocytes appears to be unaffected by an uncompromised implantation rate.” Our matched results also showed a similar live birth rate between the DEG group and the NONDEG group following fresh ET. However, the comparison is limited by several biases. First, the analyses on fresh ET cycles excluded certain types of patients, such as patients with an extremely high ovarian response or patients who failed to have day 5 blastocysts. As shown in our study, the latter may take a higher proportion in the DEG group. Second, including only fresh ET cycles omitted the contribution of surplus embryos of the cohort. As shown in previous studies (8, 18), the presence of oocyte degeneration in ICSI cycles is also associated with a decreased number of good-quality embryos. If the initial fresh ET attempt fails, the number and quality of the remaining embryos are key determinants for subsequent success.

Our study conflicted with the work of Hu et al., which suggested that the cumulative live birth rate in young women was not affected by the presence of oocyte degeneration (18).The controversial results may be caused by the different approaches of insemination (IVF vs. ICSI) or the heterogeneity of patients. The severity of male infertility might also contribute to embryo development competence (19) and therefore affect the cumulative outcome. The ICSI cohorts are expected to have more severe male infertility factors than IVF cohorts. The difference in the cumulative live birth rate between the two studies may also support the heterogeneity of the population.

In addition to the insemination protocol and male infertility, the degree of ovarian response might also affect the evaluation of the association between oocyte degeneration and cycle outcomes. Pride et al. (4) reported cycles with a higher number of oocytes correlated with the increased presence of DEG oocytes. Cinar et al. (20) reported that the oocyte degeneration rate (DEG/total oocyte) increased when more oocytes were collected per cycle, and the fertilization rate and cleavage rate in DEG cycles were significantly lower. These data suggested that DEG cases are more likely to aggregate in high responders, who are expected to have higher cumulative outcomes (21, 22). On the other hand, the presence of degenerated oocytes in the DEG patients may lead to fewer available oocytes in comparison with the NON DEG patients with similar oocyte yield. Both situations may either over- or underestimate the association between oocyte degeneration and oocyte quality. To make a better estimation of the effect of oocyte degeneration, we considered the survival oocyte number (the retrieved oocyte number minus the degenerated oocyte number) as a baseline characteristic in the PS matching method.

Atzmon et al. (7) reported that the frequency of DEG oocytes per cycle was negatively correlated with the pregnancy rate in both IVF cycles and ICSI cycles at OPU, and this study is in agreement with our results. With the GMA method, we found that the oocyte degeneration rate is negatively correlated with the cumulative live birth rate in an OPU cycle in a dose–response manner. While the occurrence of low-prevalence events might be affected by the random fluctuation of data, the dose–response association may support a causal relationship (23).

As we know, oocyte quality predominantly determines embryo quality (24–26), and oocyte morphology may affect subsequent embryo cleavage patterns and development potential (27, 28). Oocyte quality is affected by various factors like the body mass index (29), blood lipid level, and blood estrogen level (30), but the cause of oocyte degeneration was less clear. Several studies reported controversial associations between oocyte degeneration and ovarian stimulation or ovarian response (7, 20). In one study, patients with the GnRH agonist protocol had a higher risk of DEG oocytes (7), while Cinar et al. (20) reported that the GnRH antagonist protocol was correlated with more damaged oocytes. On the other hand, the E_2_ level was demonstrated to be very important during oocyte development and maturation (31); Rosen et al. (1) reported that a high level of E_2_ on the hCG day may be negatively associated with the degeneration rate in GnRH agonist protocols, while Palermo et al. (32) reported that the oocyte degeneration rate increased in patients who took more gonadotropin administration and had a lower E_2_ level on the hCG day. Factors like the ovarian stimulation protocol and high levels of E_2_ are known to affect the physiological status of women and thus affect IVF outcomes (33–36). However, with adjustment for these factors, our study suggested that the effect of oocyte degeneration in a cohort is not mediated or confounded by the physiological or cycle-based parameters. Our study may support the hypothesis that oocyte degeneration is likely a function of the inherent oocyte quality and reflects the cohort of embryo development potential.

While ICSI is absent in the conventional IVF cycles, oocyte degeneration may also be affected by technical factors, such as OPU needles and oocyte denudation pipettes. Pride et al. (4) deduced that the mechanical forces during oocyte pick-up may cause oocyte damage and affect cycle outcomes. Atzmon et al. reported that different types of needles used for OPU were associated with oocyte degeneration at OPU (7). In our center, we routinely used the 17G needle for oocyte pick-up; thus, this factor was also not discussed in this study. As for the oocyte denudation pipette, if the diameter of the stripping pipette is too small, then oocytes may be strongly compressed during striping and may result in the damage of zona pellucida and subsequent oocyte degeneration. In our center, we made a series of pipettes with different diameters before denudation, and we select the proper pipette for oocyte stripping; thus, oocyte degeneration would be widely avoided during stripping.

This study was limited by its retrospectively observational design, and the results could be screwed by unrecorded or unmeasured confounders. Although PS matching was used to minimize the confounding bias, the sample size decreased after PS matching, and the loss of unmatched samples may include biased ones; thus, we also analyzed the pre-matched data by using multivariable logistic regression analysis and got the same reassuring conclusion.

In most clinics during conventional IVF procedures, cumulus–oocyte complexes are usually incubated with spermatozoa overnight and the real oocyte structure is unknown on the oocyte pick-up day (37, 38). The oocyte morphology on the OPU day may reflect the real oocyte characteristics, without confounding the delayed effect of previous stress during denudation or OPU. Short-term insemination and early denudation provide a chance to observe oocyte degeneration of conventional IVF patients on the OPU day. Short-term insemination combined with early rescue ICSI could efficiently prevent the occurrence of total fertilization failure and got similar clinical outcomes compared with traditional IVF with overnight co-incubation of gametes (39, 40). This study was based on a cohort of patients receiving short-term insemination; caution should be taken when generalizing the conclusion to clinics using traditional IVF with overnight coincubation of gametes.

## Conclusions

In conclusion, we first reported oocyte degeneration in conventional IVF cycles on the OPU day in this study. Our results showed that the oocyte degeneration rate and degeneration cycle rate on the OPU day in conventional IVF cycles were all much lower than in ICSI cycles (2, 8). The presence of oocyte degeneration in a cohort of oocytes in conventional IVF cycles may adversely affect subsequent embryo development potential, and the cumulative live birth rate was also significantly lower in the DEG group than in the NON DEG group. These results together conferred that oocyte degeneration in conventional IVF cycles may adversely affect oocyte development potential and clinical outcomes.

## Data availability statement

The original contributions presented in the study are included in the article/**Supplementary Material**. Further inquiries can be directed to the corresponding authors.

## Ethics statement

The studies involving human participants were reviewed and approved by Ethical Committee of Medical College Xiamen University. Written informed consent for participation was not required for this study in accordance with the national legislation and the institutional requirements.

## Author contributions

LL, JLC, and JR contribute to conception and design. JHC, CY, KC, and XY contribute to the acquisition of data. LL, XJ, and ZL contributed to the analysis and interpretation of data. All authors contributed to drafting the article or revising it critically for important intellectual content. All authors contributed to the article and approved the submitted version.

## Supplementary material

Supplementary Table S1 Original data was shown in Table S1.

Supplementary Figure S1 Distributions of the distances (propensity scores) before and after PS matching were plotted in Figure S1.
