# Supplementary figures and images for: Oocyte degeneration in a cohort adversely affects clinical outcomes in conventional IVF cycles: a propensity score matching study
Source: Front Endocrinol (Lausanne). 2023 May 19;14:1164371. doi: 10.3389/fendo.2023.1164371 (PMC10235780; doi:10.3389/fendo.2023.1164371)

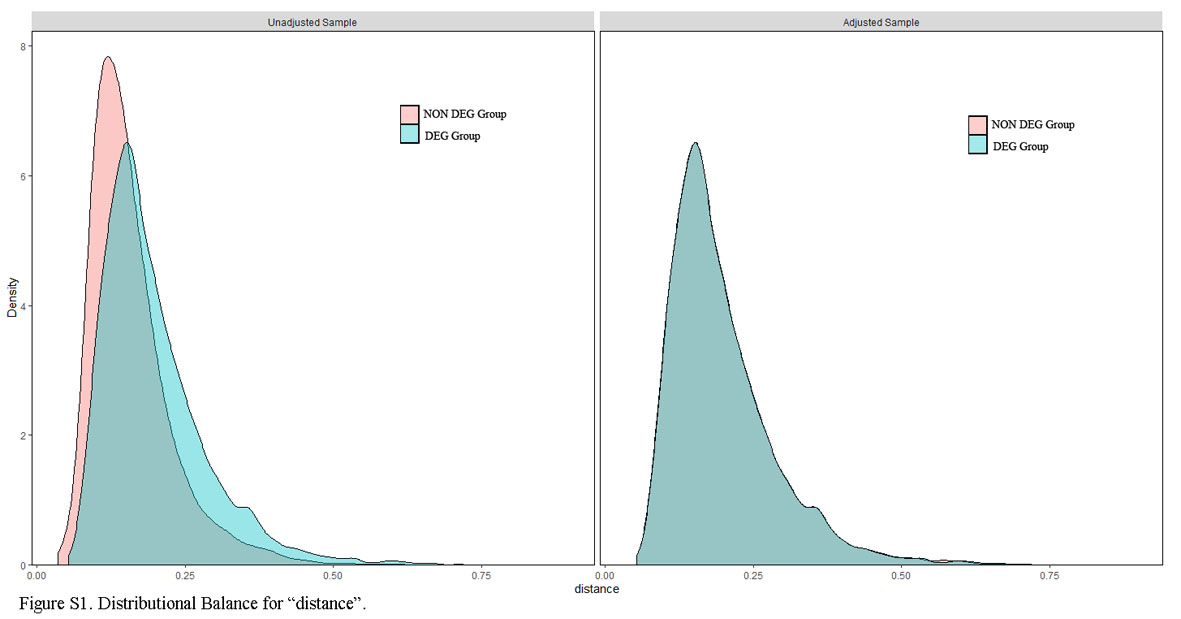

Supplement: Supplementary Figure S1 — Distributions of the distances (propensity scores) before and after PS matching were plotted in Figure S1. [file Image_1.jpeg]
